# Immuno-PET Detects Changes in Multi-RTK Tumor Cell Expression Levels in Response to Targeted Kinase Inhibition

**DOI:** 10.2967/jnumed.120.244897

**Published:** 2021-03

**Authors:** Patricia M.R. Pereira, Jalen Norfleet, Jason S. Lewis, Freddy E. Escorcia

**Affiliations:** 1 Department of Radiology, Memorial Sloan Kettering Cancer Center, New York, New York; 2 Molecular Pharmacology Program and Radiochemistry and Molecular Imaging Probes Core, Memorial Sloan Kettering Cancer Center, and Departments of Pharmacology and Radiology, Weill Cornell Medical College, New York, New York; and; 3 Molecular Imaging Program, Center for Cancer Research, National Cancer Institute, National Institutes of Health, Bethesda, Maryland

**Keywords:** immuno-PET, molecular imaging, kinases, oncology, theranostics

## Abstract

Receptor tyrosine kinase (RTK) coexpression facilitates tumor resistance due to redundancies in the phosphatidylinositol-3′-kinase/protein kinase B and KRAS/extracellular-signal–regulated kinase signaling pathways, among others. Crosstalk between the oncogenic RTK hepatocyte growth factor receptor (MET), epidermal growth factor receptor (EGFR), and human epidermal growth factor receptor 2 (HER2) are involved in tumor resistance to RTK-targeted therapies. **Methods:** In a relevant renal cell carcinoma patient–derived xenograft model, we use the ^89^Zr-labeled anti-RTK antibodies (immuno-PET) onartuzumab, panitumumab, and trastuzumab to monitor MET, EGFR, and HER2 protein levels, respectively, during treatment with agents to which the model was resistant (cetuximab) or sensitive (INC280 and trametinib). **Results:** Cetuximab treatment resulted in continued tumor growth, as well as an increase in all RTK protein levels at the tumor in vivo on immuno-PET and ex vivo at the cellular level. Conversely, after dual MET/mitogen-activated protein kinase inhibition, tumor growth was significantly blunted and corresponded to a decrease in RTK levels. **Conclusion:** These data show the utility of RTK-targeted immuno-PET to annotate RTK changes in protein expression and inform tumor response to targeted therapies.

## Visual Abstract

**Figure:**
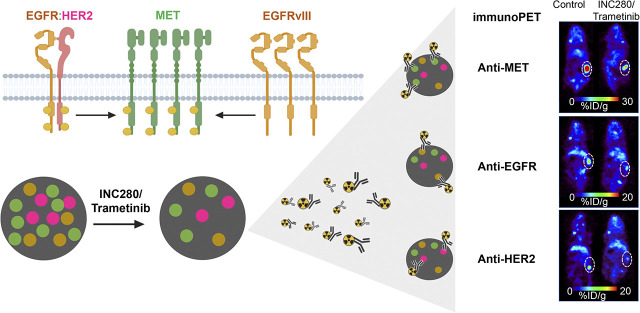

Dysregulated receptor tyrosine kinase (RTK) signaling in cancer represents an important contributor to oncogenesis and, accordingly, a valuable therapeutic and imaging target (1–3). Members of the human epidermal growth factor receptor (HER or ErbB) family of RTKs include epidermal growth factor receptor (EGFR)/ErbB1, HER2/ErbB2, HER3/ErbB3, and HER4/ErbB4 and are involved in the activation of downstream oncogenic pathways, including the KRAS/extracellular-signal–regulated kinase and phosphatidylinositol-3′-kinase/protein kinase B (PI3K-AKT) pathways (4). Activation of the ErbB pathway typically results from either *ErbB* gene amplification or activating somatic mutations, which have been described in several solid tumors (1,5). Antibodies and small-molecule inhibitors targeting the membrane domain and tyrosine kinase domains of RTKs, respectively, are used in cancer therapy and imaging (2,4,6). The EGFR-specific antibodies cetuximab and panitumumab have been widely used in the treatment of EGFR-expressing and *KRAS* wild-type colorectal cancer; and the HER2-targeting antibody trastuzumab has been successful in improving the outcomes of patients with HER2-expressing breast cancer (4,5,7). Most recently, trastuzumab–drug conjugates have been used to deliver cytotoxic drugs to breast cancers (8,9).

The hepatocyte growth factor receptor (MET, c-Met, or HGFR) is an RTK that is activated by the binding of its cognate ligand, hepatocyte growth factor (HGF) (10). High MET expression levels have been detected in several malignancies, such as breast, pancreatic, lung, bladder, and kidney cancers and gliomas (11–13). Onartuzumab is a MET-specific monoclonal antibody that inhibits activation by blocking HGF binding to MET (14). MET synergizes with members of the ErbB family of RTKs to boost cellular division and oncogenesis (15). In tumors coexpressing EGFR and MET, stimulation of EGFR enhances stability of MET protein and facilitates phosphorylation and activation of MET. MAPK appears to further enhance EGFR-dependent phosphorylation of MET (16). MET pathway activation is an important mechanism of resistance to ErbB-directed therapies (17–20). Specifically, coexpression of ErbB and MET allows PI3K/AKT oncogenic signaling to be sustained independent from ErbB downregulation or inhibition. In fact, the coexpression of several RTKs supports these acquired mechanisms of tumor resistance (21). Given the interdependence and cross-activation of oncologically relevant RTKs, determining which kinases are coexpressed in the same tumor may inform RTK-directed therapies and improve outcomes. Ex vivo immunohistochemical staining, gene amplification, and activating mutations of RTKs are critical for defining targetability with biologics or small-molecule drugs but may not represent an accurate measure of target protein availability in vivo. In fact, gene amplification and activating mutations of a certain RTK are poor predictors of response to RTK-targeted therapies (22). Methods allowing real-time, in vivo monitoring of RTK dynamics may inform treatment choice and response.

RTK-specific PET allows in vivo imaging of expression of RTKs in real time (2,3,23–27). PET imaging with RTK-targeted radiolabeled antibodies, known as immuno-PET, is a powerful method to select patients for specific therapies, can predict patient response to RTK-targeted inhibition therapy when used in the right context, and can determine the in vivo dynamics of RTKs (3,19,28). In this study, we used MET-, EGFR-, and HER2-targeted immuno-PET to detect RTK protein levels after targeted therapy in a renal cell carcinoma (RCC) patient–derived xenograft (PDX) model (29).

## MATERIALS AND METHODS

### PDXs

PDXs (collecting-duct carcinoma, an RCC subtype) were minced, mixed with Matrigel (Corning), and implanted subcutaneously in the right flank of 4- to 6-wk-old female NSG mice (Jackson Laboratories) (29). Once established, tumors were maintained and expanded by serial subcutaneous transplantation. Mouse studies were initiated once tumors reached 100–150 mm^3^ in size.

### Conjugation and Radiolabeling of Antibodies

We adhere to the nomenclature rules for radiopharmaceutical chemistry (30). Panitumumab, trastuzumab, and cetuximab were obtained from the Memorial Sloan Kettering hospital pharmacy. Onartuzumab was provided by Genentech. The antibodies were conjugated with the bifunctional chelate *p*-isothiocyanatobenzyl-desferrioxamine (DFO-Bz-NCS; Macrocyclics) and then radiolabeled with ^89^Zr in accordance with previously reported methods (28). The antibodies were conjugated with *p*-SCN-Bn-DFO in a 5:1 DFO:antibody molar ratio at 37°C for 90 min. After reaction, the conjugates were purified via a PD-10 column using Chelex (Bio-Rad) phosphate-buffered saline (0.5 g/L Chelex resin) at pH 7.4. The ^89^Zr-oxalate (supplied in 1.0 M oxalic acid at Memorial Sloan Kettering Cancer Center (28)) was neutralized to pH 7.0–7.5 with 1.0 M Na_2_CO_3_ followed by addition of the corresponding DFO–antibody conjugate in Chelex phosphate-buffered saline (pH 7.4). The mixture was incubated at 37°C for 1 h on an agitating heating block. [^89^Zr]Zr-DFO-antibody and radiochemical purity was determined by instant thin-layer chromatography, and the product was used for in vivo studies.

### PET Imaging, Biodistribution, Autoradiography Studies, and Ex Vivo Analyses

Mice were randomized into groups, and treatments were initiated (5 mice per group for biodistribution and 3 mice per group for PET imaging).

#### PET Imaging and Biodistribution Studies at Different Time Points with [^89^Zr]Zr-DFO-Onartuzumab

Mice were given [^89^Zr]Zr-DFO-onartuzumab (2.7 MBq, 15 μg, 750 μg/kg), and PET imaging was performed at 24, 48, 72, 96, and 120 h after injection. Biodistribution studies were performed at 24, 72, and 120 h after injection of [^89^Zr]Zr-DFO-onartuzumab. Additional biodistribution studies were performed on PDX-bearing mice that were blocked with a 25-fold mass excess of unlabeled onartuzumab 48 h before injection of [^89^Zr]Zr-DFO-onartuzumab to confirm target specificity.

#### PET Imaging and Biodistribution Studies After Treatments

INC280, a MET-selective tyrosine kinase inhibitor, and trametinib, a mitogen-activated protein kinase kinase (MEK) inhibitor, were obtained from the Neal Rosen research group at Memorial Sloan Kettering. PDX-bearing mice were treated with cetuximab or with INC280 and trametinib following previously reported methods (29). Briefly, cetuximab was intravenously administered (50 mg/kg) twice a week for 10 d. INC280 (10 mg/kg) and trametinib (1.5 mg/kg) were orally administered daily for 10 d. [^89^Zr]Zr-DFO-onartuzumab (2.7 MBq, 15 μg), [^89^Zr]Zr-DFO-panitumumab (11.0 MBq, 50 μg), and [^89^Zr]Zr-DFO-trastuzumab (8.14 MBq, 80 μg) were administered by tail vein injection on day 10. PET imaging and biodistribution studies were performed 120 h after injection of [^89^Zr]Zr-DFO-antibodies.

PET imaging, acute biodistribution, and autoradiography studies were performed at 120 h after intravenous injection of [^89^Zr]Zr-DFO-antibody according to previously reported methods (24,25,31).

### Western Blot Analysis

Whole-protein extracts from PDXs were obtained in radioimmunoprecipitation assay buffer as previously described (25). After electrophoresis and transfer to nitrocellulose membranes (IB23001; Thermo Fisher Scientific), the blots were incubated in 5% w/v bovine serum albumin (A7030; Sigma) in Tris-buffered saline with polysorbate (9997S; Cell Signaling Technology) and probed with mouse anti-β-actin, 1:20,000 (A1978; Sigma); rabbit anti-HER2, 1:800 (ab131490; Abcam); rabbit anti-phosphorylated HER2 (anti-pHER2), 1:800 (ab53290; Abcam); rabbit anti-EGFR, 1:1,000 (ab52894; Abcam); rabbit anti-MET, 1:1,000 (ab51067; Abcam); and anti-phosphorylated MET (anti-pMET), 1:1,000 (ab68141; Abcam). After antibody incubation and washing, the membranes were incubated with the secondary antibodies IRDye 800CW anti-rabbit (925-32211) or anti-mouse (925-32210) IgG, 1:15,000 (LI-COR Biosciences), and imaged on the Odyssey Infrared Imaging System (LI-COR Biosciences), followed by densitometric analysis using Fiji software (https://imagej.net/Fiji) (32).

### ELISA for Human HGF Protein Levels in Serum and Tumor Homogenates of PDX-Bearing Mice

The human HGF ELISA kit (KAC2211; Invitrogen) was used for quantitative determination of human HGF in serum and tumor homogenates of PDX-bearing mice as previously described (27).

### Statistical Analysis

Data are expressed as mean ± SEM. Groups were compared using the Student *t* test.

### Study Approval

All animals were treated according to the guidelines approved by the Research Animal Resource Center and Institutional Animal Care and Use Committee at Memorial Sloan Kettering Cancer Center. PDX models were established, by the Antitumor Assessment Core, from tumor specimens collected under an institutional review board protocol approved by the same committee.

## RESULTS

### MET-Targeted Immuno-PET Detects MET-Expressing RCC PDXs

Previous preclinical studies have demonstrated the potential of ^89^Zr-labeled onartuzumab to image MET-expressing tumors (23,26,33). To determine the ability of ^89^Zr-labeled onartuzumab to image a MET-overexpressing tumor model sensitive to MET-targeted therapy, our preclinical studies used the MET-overexpressing human RCC PDX (29). HGF, the MET ligand, was detected by ELISA in the plasma and tumors of immunodeficient NOD-SCID γ (NSG) mice implanted subcutaneously with RCC PDX, confirming autocrine production of HGF in this model (Supplemental Table 1; supplemental materials are available at http://jnm.snmjournals.org).

**FIGURE 1.**
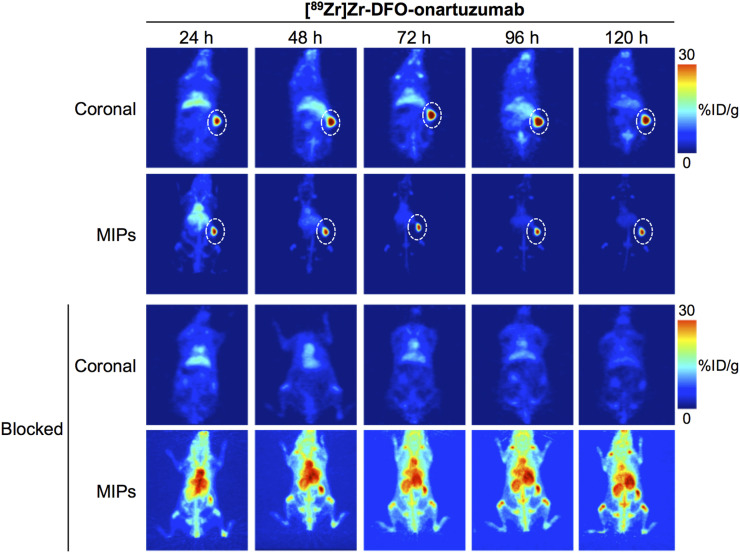
Representative maximum-intensity-projection (MIP) and coronal ^89^Zr-labeled onartuzumab PET images at 24, 48, 72, 96, and 120 h after injection of [^89^Zr]Zr-DFO-onartuzumab without and with unlabeled onartuzumab blocking in NSG mice bearing subcutaneous MET-overexpressing PDX RCC. [^89^Zr]Zr-DFO-onartuzumab (2.7 MBq, 15 μg, 750 μg/kg) was administered by tail vein injection. Blocking experiments were performed by administering 25-fold mass excess of unlabeled onartuzumab 48 h before injection of [^89^Zr]Zr-DFO-onartuzumab.

**FIGURE 2.**
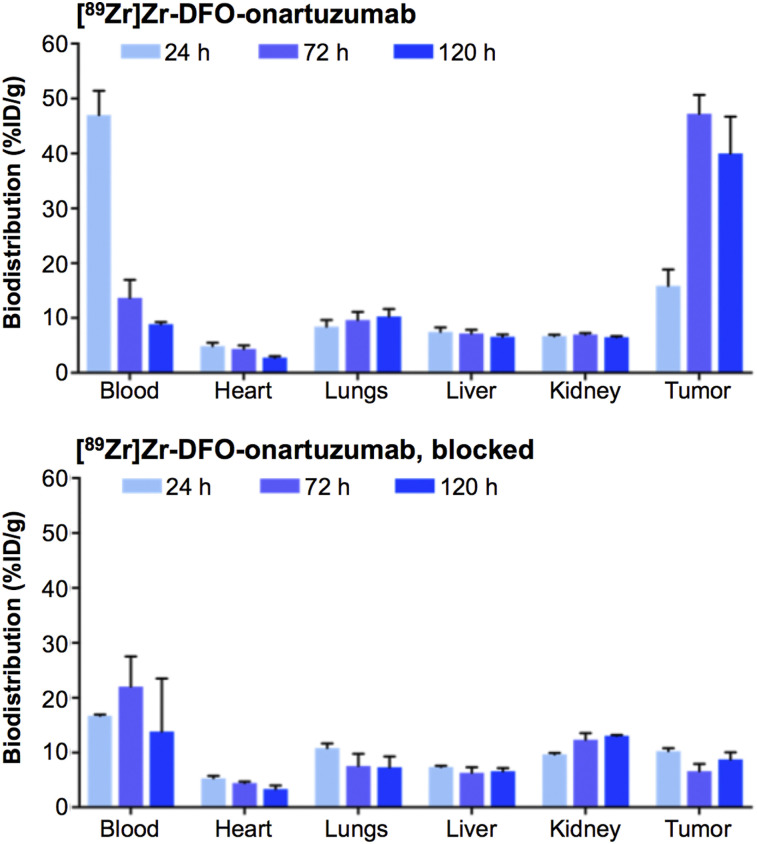
Biodistribution at 24, 72, and 120 h after injection of [^89^Zr]Zr-DFO-onartuzumab without and with unlabeled-onartuzumab blocking in NSG mice bearing subcutaneous MET-overexpressing PDX RCC. [^89^Zr]Zr-DFO-onartuzumab (2.7 MBq, 15 μg, 750 μg/kg) was administered by tail vein injection. Blocking experiments were performed by administration of 25-fold mass excess of unlabeled onartuzumab 48 h before injection of [^89^Zr]Zr-DFO-onartuzumab.

In vivo PET imaging studies with ^89^Zr-labeled onartuzumab confirmed excellent target localization in subcutaneous RCC PDXs (Fig. 1). Ex vivo biodistribution of the radioimmunoconjugate demonstrated a gradual accumulation into the MET-positive tumors between 24 and 72 h. Percentage injected dose per gram (%ID/g) was 15.9 ± 7.3 at 24 h and 47.3 ± 8.3 at 72 h (Fig. 2; Supplemental Fig. 1; Supplemental Table 2). Tumoral uptake of ^89^Zr-labeled onartuzumab peaked at 72 h and persisted to at least 120 h (47.3 ± 8.3 %ID/g at 72 h and 40.0 ± 16.4 %ID/g at 120 h; Fig. 2; Supplemental Fig. 1; Supplemental Table 2). Furthermore, the accumulation of ^89^Zr-labeled onartuzumab in RCC PDXs could be blocked using coinjection of a 25-fold mass excess of unlabeled onartuzumab antibody, confirming target specificity (Figs. 1 and 2; Supplemental Figs. 1 and 2). Autoradiography analysis of tumors from mice administered ^89^Zr-labeled onartuzumab or ^89^Zr-labeled onartuzumab in the presence of an excess of unlabeled onartuzumab confirmed our findings from in vivo PET imaging and ex vivo biodistribution (Supplemental Fig. 3).

Taken together, these studies support that ^89^Zr-labeled onartuzumab can image MET-overexpressing RCC PDXs and detect MET protein levels in vivo.

### Immuno-PET Detects Changes in RTK Protein Levels After RTK-Targeted Therapy

Given that RTK coactivation plays an important role in tumor response to RTK-targeted therapy (19,29,34), we sought to use molecular imaging to understand and visualize the interplay between MET, EGFR, and HER2 receptor dynamics in our PDX model, which expresses all 3 receptors. Previous studies have demonstrated that the MET-overexpressing RCC PDX harbors activating BRAF (G466A and D594N) mutations and is sensitive to both MET (INC280, or capmatinib) and MEK (trametinib) inhibition. Although noted to have significant EGFR expression, the RCC PDX was observed to have resistance to cetuximab treatment, likely due to RAS-RAF pathway activation (29). We exploited the known resistance to cetuximab of this model, and the sensitivity to combined INC280 and trametinib, to ascertain whether we could use immuno-PET to noninvasively assess response to therapy.

**FIGURE 3.**
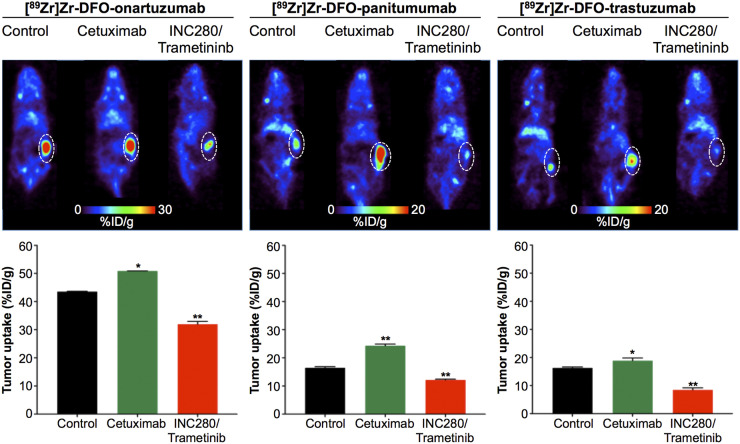
Representative coronal PET images and tumor uptake as determined by ex vivo biodistribution at 120 h after injection of [^89^Zr]Zr-DFO-onartuzumab, [^89^Zr]Zr-DFO-panitumumab, or [^89^Zr]Zr-DFO-trastuzumab in NSG mice bearing subcutaneous PDX RCC and treated with saline (control), cetuximab, or both INC280 and trametinib. Cetuximab was intravenously administered (50 mg/kg of body weight) twice weekly for 10 d. INC280 (10 mg/kg) and trametinib (1.5 mg/kg) were orally administered daily for 10 d. [^89^Zr]Zr-DFO-onartuzumab (2.7 MBq, 15 μg, 750 μg/kg), [^89^Zr]Zr-DFO-panitumumab (11.0 MBq, 50 μg, 2.5 mg/kg), and [^89^Zr]Zr-DFO-trastuzumab (8.14 MBq, 80 μg, 4 mg/kg) were administered by tail vein injection on day 10 (Supplemental Fig. 4). PET images and biodistribution studies were performed at 120 h after injection of ^89^Zr-labeled antibodies. *P < 0.05. **P < 0.01. *P* values are based on Student *t* test and compared with control.

In our in vivo studies, we used ^89^Zr-labeled anti-HER2 (trastuzumab), ^89^Zr-labeled anti-EGFR (panitumumab), or ^89^Zr-labeled anti-MET (onartuzumab) antibodies to monitor RTK protein levels in RCC PDXs after targeted therapy. Mice were treated with cetuximab or with combined INC280 and trametinib for 10 d (Supplemental Fig. 4). Cetuximab treatment did not alter tumor volume (Supplemental Fig. 5), consistent with previous reports that this PDX is resistant to cetuximab (29). Mice treated with combined INC280 and trametinib showed significant tumor growth inhibition over 10 d (Supplemental Fig. 5). Longitudinal PET imaging (Fig. 3) and ex vivo biodistribution (Fig. 3; Supplemental Figs. 6–8; Supplemental Tables 2–4) at 120 h after injection of ^89^Zr-labeled anti-RTK antibodies demonstrated a significant difference in tumor uptake of the radioimmunoconjugate between control and cetuximab-treated tumors and between INC280 and trametinib-treated tumors (Fig. 3; Supplemental Figs. 6–8). Control tumors had an ^89^Zr-labeled trastuzumab uptake of 16.33 ± 0.32 %ID/g, an ^89^Zr-labeled panitumumab uptake of 16.51 ± 0.40 %ID/g, and an ^89^Zr-labeled onartuzumab uptake of 43.52 ± 0.12 %ID/g. Tumors treated with cetuximab showed higher uptake of tracers than did control treated tumors: 18.90 ± 0.95 %ID/g for ^89^Zr-labeled trastuzumab, 24.33 ± 0.58 %ID/g for ^89^Zr-labeled panitumumab, and 50.88 ± 0.01 %ID/g for ^89^Zr-labeled onartuzumab. In tumors of mice treated with a combination of INC280 and trametinib, we observed a lower tumor uptake than that seen in control treated tumors: 8.53 ± 0.68 %ID/g for ^89^Zr-labeled trastuzumab, 12.18 ± 0.28 %ID/g for ^89^Zr-labeled panitumumab, and 31.97 ± 0.95 %ID/g for ^89^Zr-labeled onartuzumab.

These results demonstrate the potential of immuno-PET to visualize changes in MET, EGFR, and HER2 during RTK-targeted therapy in vivo. Additionally, our results show an increase in RTK protein levels during therapy with cetuximab. Therapy with combined MET/MEK inhibition decreases anti-RTK antibody accumulation in tumors from RCC PDXs, suggesting that a decrease in the plasma membrane levels of MET, EGFR, and HER2 corresponds to therapeutic benefit.

### In Vivo RTK-Targeted Immuno-PET Correlates with Ex Vivo Changes in MET, EGFR, and HER2 Protein Levels

**FIGURE 4.**
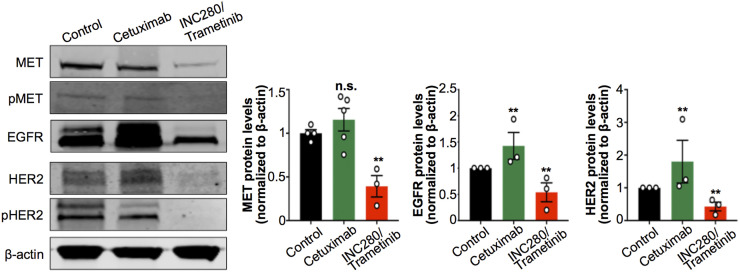
Protein expression and activation of MET, EGFR, and HER2 as analyzed and quantified by Western blot of tumors from NSG mice bearing subcutaneous PDX RCC and treated with saline (control), cetuximab, or combined INC280 and trametinib. Cetuximab was intravenously administered (50 mg/kg of body weight) twice weekly for 10 d. INC280 (10 mg/kg) and trametinib (1.5 mg/kg) were orally administered daily for 10 d. Tumors were collected on day 15 (Supplemental Fig. 4), and protein expression was analyzed by Western blot. **P < 0.01, based on Student *t* test and compared with control.

Having found changes in tumor uptake of ^89^Zr-radiolabeled antibodies after treatment with cetuximab or with INC280 and trametinib, we next investigated changes in MET, EGFR, and HER2 protein levels at the cellular level. We performed immunoblot studies of tumor digests to detect changes in MET, pMET, EGFR, HER2, and pHER2 on cetuximab or INC280 and trametinib treatments. We observed a decrease in MET, pMET, EGFR, HER2, and pHER2 in tumors of mice treated with INC280 and trametinib (Fig. 4). Conversely, tumors of mice treated with cetuximab showed a compensatory increase in MET, EGFR, and HER2 protein when compared with control mice (Fig. 4). The changes observed in RTK protein levels in tumors of mice treated with cetuximab or with INC280 and trametinib were consistent with those observed for ^89^Zr-labeled antibody tumor uptake and collectively suggest a mechanism of resistance of this EGFR-expressing PDX to cetuximab (Fig. 3; Supplemental Fig. 5). The changes observed in RTK/pRTK in Figure 4 are concordant with previous observations of downstream oncogenic signaling in RCC PDX cells using RTK arrays (29). In the PDX cells, ERK signaling is sensitive to MET inhibition but not to cetuximab. Additionally, the combination of MET and MEK inhibition (INC280 and trametinib) blocks ERK signaling and cell growth.

These results confirm that in vivo RTK-targeted immuno-PET correlates with changes in MET, EGFR, and HER2 protein at the cellular level and may noninvasively annotate early response to targeted therapy.

## DISCUSSION

Precision medicine involves identification of certain gene mutations and expressions, as well as characterization of proteomic and epigenetic features, that contribute individual tumor signatures. However, the spatial distribution, dynamics, and heterogeneity of RTKs can influence the response of tumor populations to RTK-targeted inhibitors. RTK gene amplification, enhanced protein expression, and specific mutations result in RTK dimerization and clustering at the cell membrane that can trigger ligand-independent or kinase-independent activation of RTKs of the same family or collaborating RTKs. RTK crosstalk and activation maintain oncogenic signaling networks, including RAS-ERK and PI3K-AKT. These processes have immediate effects on the way that tumors respond to RTK-targeted therapy, such as cetuximab, an antibody that competes with the EGF ligand for binding to EGFR (7). Indeed, tumor response to monotherapy with EGFR-targeted antibodies is relatively low (∼10%), with a significant yet modest improvement in overall survival restricted to patients with KRAS wild-type tumors (35). Crosstalk between EGFR and other members of the HER family or unrelated RTKs, as mediated by coexpression of RTKs in the same tumor, neutralize the growth-inhibitory properties of EGFR-targeted therapies due to redundancy in mechanisms of PI3K-AKT and RAS-ERK activation (16–18,36). Preclinical and clinical studies with combinatorial RTK inhibition have been explored in tumors for which MET activation occurs as a mechanism of acquired resistance to EGFR-targeted therapies (4). These studies suggest an abrogation of resistance by inactivating multiple RTKs upstream from key oncogenic nodes and support the idea that patients could benefit from those combinatorial strategies. Amplification of the *MET* gene, along with subsequent protein activation, has been observed in RCC, and MET overexpression is known to be a negative prognostic biomarker (12,37–39). Given that RTK coactivation plays an important role in tumor response to targeted therapy (19,29,34), we sought to use molecular imaging to understand and assess the interplay between, and the dynamics of, MET, EGFR, and HER2.

We extended our prior work using immuno-PET for imaging RTK membrane dynamics and crosstalk, and we assessed its utility in an RCC PDX model known to express MET, EGFR, and HER2 (29). Although the PDX used in our studies does not harbor activating MET mutations or increased MET copy number, it demonstrates sensitivity to inhibition of MET (INC280) and MEK (trametinib) as monotherapy or in combination (29). These findings underscore that MET overexpression alone may identify tumors that may be susceptible to RTK-targeted therapy in the appropriate setting. The RCC PDX model harbors RAS-pathway activation via BRAF mutations (G466A and D594N), with corresponding ERK signaling activation, which may partially explain its insensitivity to cetuximab treatment (29). We exploited this differential response to targeted therapy and confirmed that immuno-PET could noninvasively annotate resistance and response to treatment.

We found that immuno-PET with the ^89^Zr-labeled anti-RTK antibodies onartuzumab, trastuzumab, and panitumumab can serve as a sensor to MET, EGFR, and HER2, respectively, in vivo. Tumors of animals treated with combined MET/MEK inhibition showed growth suppression, as expected, and exhibited lower uptake on quantitative MET immuno-PET. These findings corresponded to changes at the cellular level, where we observed decreased MET protein levels and MET phosphorylation in tumors. Interestingly, uptake of EGFR and HER2 immuno-PET tracers was also lower in tumors treated with combined MET/MEK inhibition, which also corresponded to decreased total levels of HER2, EGFR, and pHER2 in tumor digests, suggesting that treatment may also interfere with the MET-HER2 cross activation (40).

Conversely, tumors of animals treated with cetuximab showed increased pan-RTK immuno-PET tracer uptake, which was mirrored by protein levels of tumor digests. These increases in MET, EGFR, and HER2 protein levels might represent a possible mechanism of resistance of this EGFR-expressing PDX to cetuximab and are consistent with previous reports of cetuximab-resistant cells arising from alterations in HER trafficking and protein degradation (41).

Admittedly, implementing 3 separate immuno-PET tracers with the long-lived ^89^Zr to provide readouts of treatment response stretches the limits of clinical feasibility. Engineered therapeutic antibodies targeting multiple RTKs may present a possible immuno-PET approach to visualizing changes in RTKs as a class after treatment with kinase-directed therapies. Lower-molecular-weight targeting biomolecules and radionuclides with shorter blood and physical half-lives, respectively, may offer time frames more congruent with current clinical workflows and should be investigated. Additionally, visualizing cellular signaling nodes, which are activated by several RTKs, as was recently performed by Pratt et al. using ^124^I-labeled trametinib is being investigated and could achieve similar treatment-response readouts (42).

## CONCLUSION

Our results highlight the potential of RTK-targeted immuno-PET as a functional sensor of plasma membrane levels of RTKs in tumors and, importantly, identified tumor features associated with treatment response and resistance to systemic therapy. Such data can inform RTK-directed therapies, cellular therapy (e.g., chimeric antigen receptor T cells), and biologics (e.g., anti-RTK antibodies, antibody–drug conjugates, and targeted molecular radiotherapy), for example. Studies to better define specific circumstances in which RTK-targeted immuno-PET can annotate genetic and proteomic features of tumors associated with sensitivity or resistance to targeted therapies *ab initio* are ongoing.

## DISCLOSURE

Onartuzumab was provided by Genentech. The Radiochemistry and Molecular Imaging Probe Core and the Antitumor Assessment Core were supported by NIH grant P30 CA08748. This study was supported in part by the Geoffrey Beene Cancer Research Center of MSKCC (Jason Lewis), NIH NCI grant R35 CA232130 (Jason Lewis), NIH NCI grant ZIA BC 011800 (Freddy Escorcia), Mr. William H. and Mrs. Alice Goodwin and the Commonwealth Foundation for Cancer Research, and the Center for Experimental Therapeutics of Memorial Sloan Kettering Cancer Center. Freddy Escorcia is supported by the American Board of Radiology Leonard B. Holman Research Pathway and the Clinical Investigator Development Program of NCI and NIH. Patricia Pereira is supported by the Tow Foundation Postdoctoral Fellowship from the MSKCC Center for Molecular Imaging and Nanotechnology and the Alan and Sandra Gerry Metastasis and Tumor Ecosystems Center of MSKCC. No other potential conflict of interest relevant to this article was reported.

**KEY POINTS**

**QUESTION:** Can functional imaging with immuno-PET assess changes in protein levels of RTKs of tumors?
**PERTINENT FINDINGS:** Indeed, immuno-PET can visualize RTKs, reflects cellular protein levels, and can detect post–targeted-therapy RTK changes that correspond to sensitivity to treatment.
**IMPLICATIONS FOR PATIENT CARE:** Immuno-PET imaging of oncogenic RTKs can serve as a noninvasive biomarker for treatment stratification and can provide early insights on tumor response to target therapy.
