# Supplementary material for: Immuno-PET Detects Changes in Multi-RTK Tumor Cell Expression Levels in Response to Targeted Kinase Inhibition
Source: J Nucl Med. 2021 Mar;62(3):366–71. doi: 10.2967/jnumed.120.244897 (PMC8049345; doi:10.2967/jnumed.120.244897)
Supplement: Supplementary file 1 [file jnm244897SupplementaryData.pdf]

Supplemental Table 1. HGF in the tumor and plasma of NSG mice bearing subcutaneous PDX RCC as determined by an ELISA assay.

**HGF (pg/ml)**		
	Tumor	Plasma
24h	626.5±42.2	417.7±18.8
48h	588.8±26.4	404.7±1.89
72h	645.3±69.3	446.2±9.66

Supplemental Table 2. Biodistribution at 120 h p.i. of [^{89}Zr]Zr-DFO-onartuzumab in NSG mice bearing subcutaneous PDX RCC and treated with saline (control), cetuximab or a combination of INC280 and trametinib (labeled as INC280/ trametinib). Cetuximab was intravenously administered (50 mg/kg of mice) twice a week for 10 days. INC280 (10 mg/kg of mice) and trametinib (1.5 mg/kg of mice) were orally administered daily for 10 days. [^{89}Zr]Zr-DFO-onartuzumab (2.7 MBq, 15µg) was administrated by tail vein injection on day 10 (*see Fig.* ***SI 4***). Biodistribution studies were performed at 120 h p.i. of [^{89}Zr]Zr-DFO-onartuzumab.

	**Control (%ID/g)**			**INC280/Trametinib (%ID/g)**			**Cetuximab (%ID/g)**		
	Mean	S.D.	N	Mean	S.D.	N	Mean	S.D.	N
**Blood**	7.44	0.68	4	8.10	1.03	4	6.83	1.27	4
**Heart**	3.00	0.56	4	3.32	0.64	4	2.77	0.56	4
**Liver**	3.34	0.49	4	2.57	0.26	4	3.36	0.97	4
**Lungs**	5.54	0.69	4	6.03	1.79	4	5.22	2.12	4
**Stomach**	1.12	0.29	4	0.92	0.13	4	0.80	0.38	4
**Spleen**	3.82	0.92	4	4.03	2.28	4	3.84	1.72	4
**Pancreas**	0.96	0.12	4	1.49	0.59	4	0.90	0.21	4
**Small Intestine**	1.10	0.08	4	1.23	0.10	4	1.17	0.31	4
**Large Intestine**	1.30	0.14	4	0.99	0.21	4	1.09	0.42	4
**Kidneys**	6.59	0.33	4	6.59	1.51	4	5.01	0.39	4
**Skin**	3.31	0.23	4	3.58	0.04	4	3.26	0.70	4
**Muscle**	0.78	0.16	4	1.03	0.23	4	0.85	0.84	4
**Bone**	2.88	0.65	4	3.93	0.41	4	6.37	1.56	4
**Tail**	2.03	0.80	4	2.44	1.44	4	1.94	0.40	4
**Tumor**	43.5	9.12	4	31.9	8.95	4	50.9	12.0	4

Supplemental Table 3. Biodistribution at 120 h p.i. of [[^{89}Zr]Zr-DFO-panitumumab in NSG mice bearing subcutaneous PDX RCC and treated with saline (control), cetuximab or a combination of INC280 and trametinib (labeled as INC280/ trametinib). Cetuximab was intravenously administered (50 mg/kg of mice) twice a week for 10 days. INC280 (10 mg/kg of mice) and trametinib (1.5 mg/kg of mice) were orally administered daily for 10 days. [^{89}Zr]Zr-DFO-panitumumab (11.0 MBq, 50 µg protein) was administered by tail vein injection on day 10 (*see Fig.* ***SI 4***). Biodistribution studies were performed at 120 h p.i. of [^{89}Zr]Zr-DFO-panitumumab.

	**Control (%ID/g)**			**INC280/Trametinib (%ID/g)**			**Cetuximab (%ID/g)**		
	Mean	S.D.	N	Mean	S.D.	N	Mean	S.D.	N
**Blood**	8.05	3.69	4	13.5	1.54	4	14.5	1.98	4
**Heart**	3.63	3.05	4	3.56	0.90	4	3.38	1.21	4
**Liver**	5.76	1.03	4	4.52	0.70	4	6.33	0.93	4
**Lungs**	2.65	1.72	4	6.77	0.91	4	6.70	1.26	4
**Stomach**	0.91	0.37	4	0.82	0.16	4	1.00	0.07	4
**Spleen**	9.64	1.02	4	6.31	1.54	4	7.38	1.42	4
**Pancreas**	0.87	0.15	4	1.32	0.32	4	0.92	0.51	4
**Small Intestine**	1.07	0.24	4	1.02	0.15	4	2.41	1.98	4
**Large Intestine**	1.57	0.90	4	0.90	0.10	4	1.32	0.46	4
**Kidneys**	2.47	1.17	4	2.67	0.41	4	4.13	0.43	4
**Skin**	3.35	0.88	4	2.96	0.31	4	2.34	1.26	4
**Muscle**	0.44	0.21	4	0.75	0.13	4	0.72	0.22	4
**Bone**	4.49	1.82	4	2.91	0.64	4	5.24	2.05	4
**Tail**	1.30	0.19	4	2.83	1.17	4	3.48	3.25	4
**Tumor**	16.5	4.40	4	12.2	1.28	4	24.3	5.58	4

Supplemental Table 4. Biodistribution at 120 h p.i. of [^{89}Zr]Zr-DFO-trastuzumab in NSG mice bearing subcutaneous PDX RCC and treated with saline (control), cetuximab or a combination of INC280 and trametinib (labeled as INC280/ trametinib). Cetuximab was intravenously administered (50 mg/kg of mice) twice a week for 10 days. INC280 (10 mg/kg of mice) and trametinib (1.5 mg/kg of mice) were orally administered daily for 10 days. [^{89}Zr]Zr-DFO-trastuzumab (8.14 MBq, 80 μg protein) was administered by tail vein injection on day 10 (*see Fig.* ***SI 4***). Biodistribution studies were performed at 120 h p.i. of [^{89}Zr]Zr-DFO-trastuzumab.

	**Control (%ID/g)**			**INC280/Trametinib (%ID/g)**			**Cetuximab (%ID/g)**		
	Mean	S.D.	N	Mean	S.D.	N	Mean	S.D.	N
**Blood**	5.48	3.52	4	9.05	6.85	4	2.88	0.40	4
**Heart**	2.17	2.63	4	2.49	0.50	4	1.20	0.06	4
**Liver**	5.72	1.40	4	3.86	0.12	4	4.97	0.41	4
**Lungs**	3.13	3.09	4	3.90	0.57	4	2.71	0.20	4
**Stomach**	0.79	0.48	4	0.67	0.06	4	0.81	0.07	4
**Spleen**	13.9	4.44	4	7.78	4.39	4	10.8	5.05	4
**Pancreas**	0.91	0.75	4	0.90	0.24	4	1.01	0.59	4
**Small Intestine**	2.01	0.75	4	1.83	0.16	4	2.82	0.04	4
**Large Intestine**	1.04	0.17	4	0.76	0.34	4	1.24	0.18	4
**Kidneys**	2.41	1.06	4	1.79	0.63	4	1.84	0.12	4
**Skin**	3.96	1.45	4	2.47	0.28	4	1.91	0.24	4
**Muscle**	0.43	0.15	4	0.51	0.22	4	0.35	0.12	4
**Bone**	5.88	1.26	4	4.34	1.29	4	5.81	1.35	4
**Tail**	1.61	0.97	4	1.30	0.20	4	2.81	2.28	4
**Tumor**	16.33	0.32	4	8.53	0.68	4	18.90	0.95	4

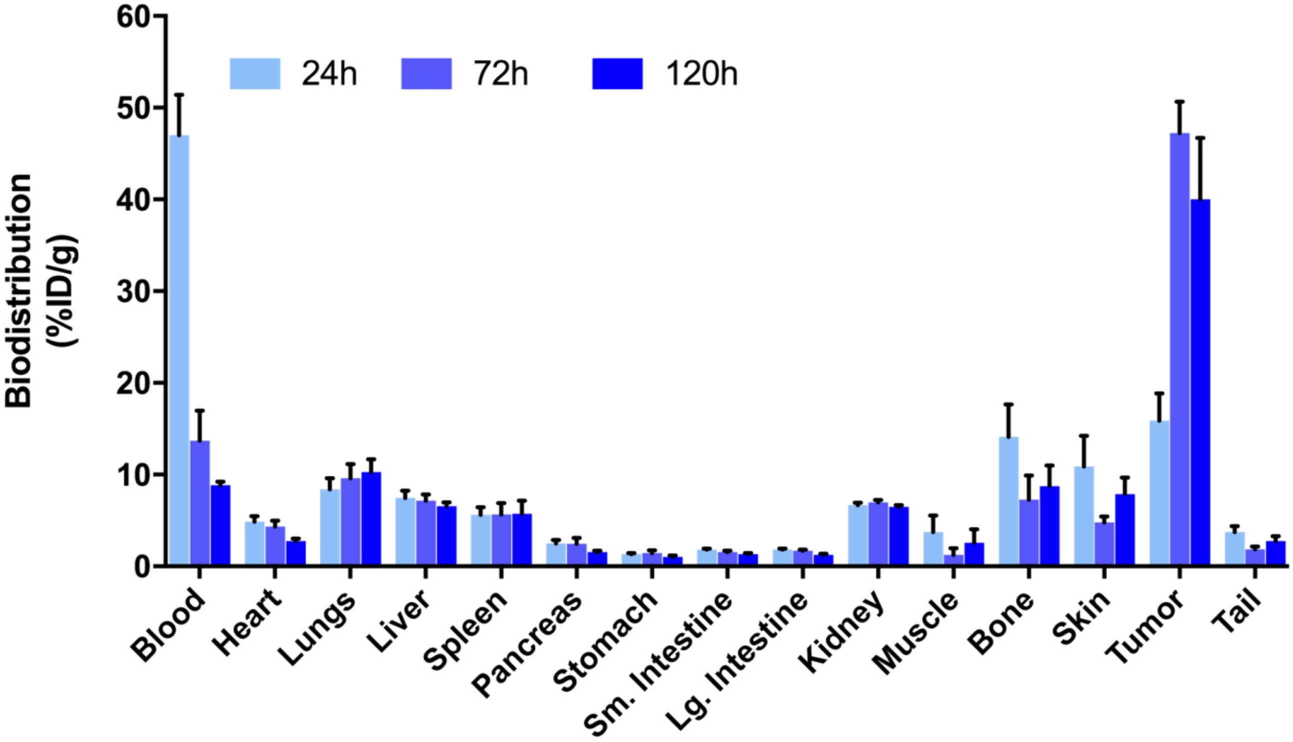

Supplemental Figure 1. Biodistribution at 24, 72, and 120 h p.i. of [^{89}Zr]Zr-DFO-onartuzumab in NSG mice bearing subcutaneous PDX RCC. [^{89}Zr]Zr-DFO-onartuzumab (2.7 MBq, 15μg) was administered by tail vein injection. %ID/g, percentage of injected dose per gram.

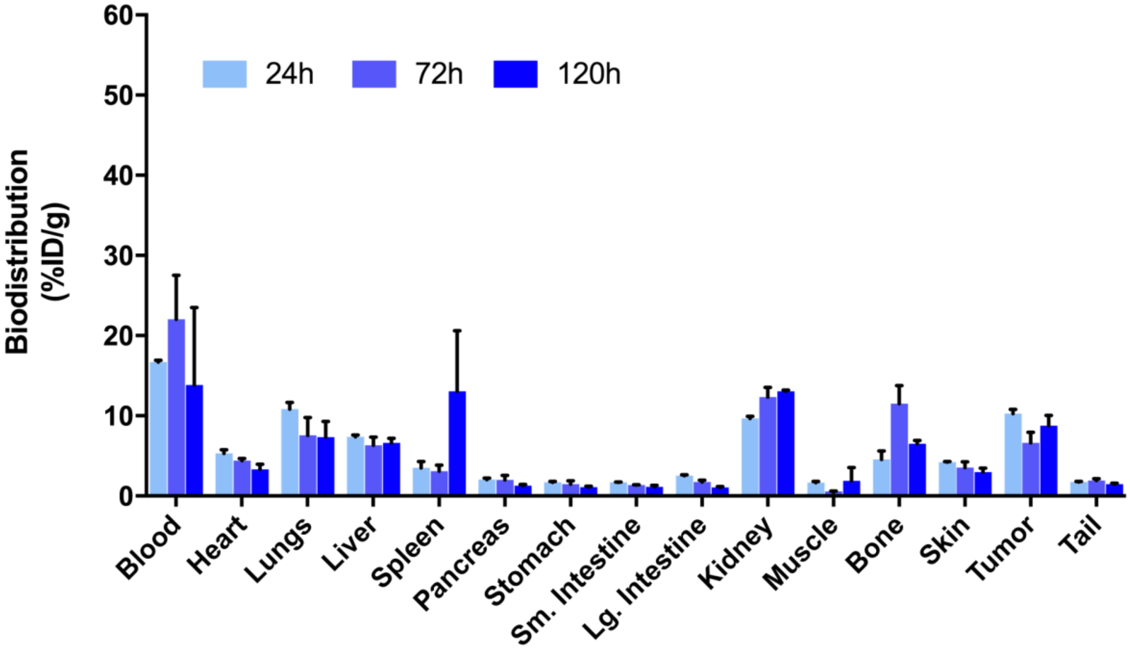

Supplemental Figure 2. Biodistribution at 24, 72, and 120 h p.i. of [^{89}Zr]Zr-DFO-onartuzumab with unlabeled onartuzumab blocking in NSG mice bearing subcutaneous PDX RCC. [^{89}Zr]Zr-DFO-onartuzumab (2.7 MBq, 15µg) was administered by tail vein injection. Blocking experiments were performed by administration of a 25-fold mass excess of unlabeled onartuzumab 48 h prior to injection of [^{89}Zr]Zr-DFO-onartuzumab. %ID/g, percentage of injected dose per gram.

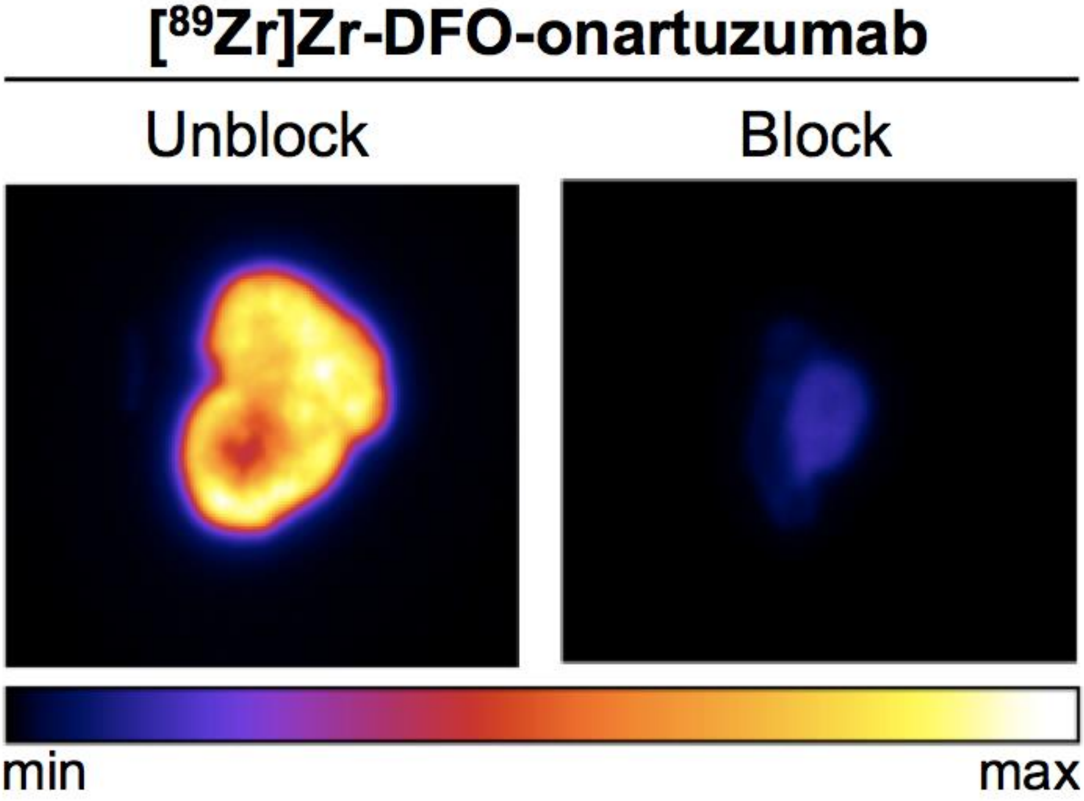

Supplemental Figure 3. Autoradiography at 120 h p.i. of [^{89}Zr]Zr-DFO-onartuzumab without and with unlabeled onartuzumab blocking in NSG mice bearing subcutaneous PDX RCC. [^{89}Zr]Zr-DFO-onartuzumab (2.7 MBq, 15µg) was administered by tail vein injection. Blocking experiments were performed by administration of a 25-fold mass excess of unlabeled onartuzumab 48 h prior to injection of [^{89}Zr]Zr-DFO-onartuzumab.

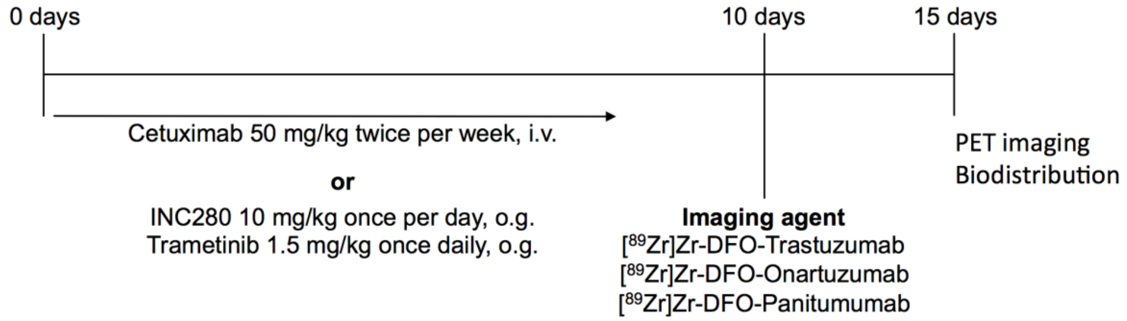

Supplemental Figure 4. Schematic diagram of PDX RCC bearing mice treated with saline (control), cetuximab or a combination of INC280 and trametinib (labeled as INC280/ trametinib) and imaged with ^{89}Zr-labeled anti-HER2 (trastuzumab), ^{89}Zr-labeled anti-EGFR (panitumumab) or ^{89}Zr-labeled anti-MET (onartuzumab) antibodies. Cetuximab was intravenously administered (50 mg/kg of mice) twice a week for 10 days. INC280 (10 mg/kg of mice) and trametinib (1.5 mg/kg of mice) were orally administrated daily for 10 days. [^{89}Zr]Zr-DFO-onartuzumab (2.7 MBq, 15µg), [^{89}Zr]Zr-DFO-panitumumab (11.0 MBq, 50 µg protein), and [^{89}Zr]Zr-DFO-trastuzumab (8.14 MBq, 80 µg protein) were administered by tail vein injection on day 10. PET images, biodistribution, and Western blot analyses were performed at 120 h p.i. of ^{89}Zr-labeled antibodies.

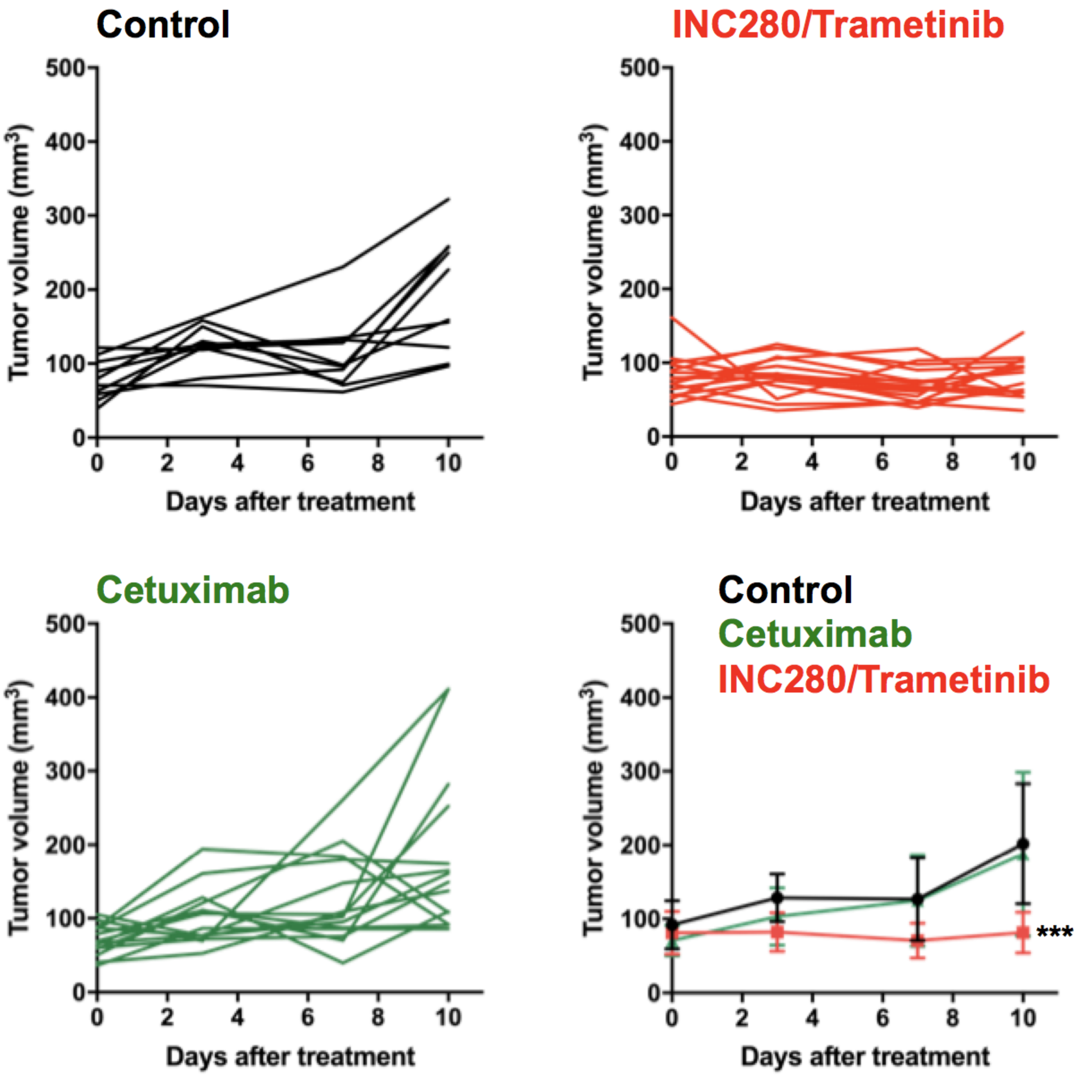

Supplemental Figure 5. Tumor volumes (mm^3) as a function of time in control (saline), cetuximab, and a combination of INC280 and trametinib (labeled as INC280/ trametinib) treated NSG mice bearing subcutaneous PDX RCC tumors (n = 10 mice per group). Cetuximab was intravenously administered (50 mg/kg of mice) twice a week for 10 days. INC280 (10 mg/kg of mice) and trametinib (1.5 mg/kg of mice) were orally administered daily for 10 days. $^{***}P < 0.001$ based on a Student's *t*-test and compared with control or cetuximab at day 10.

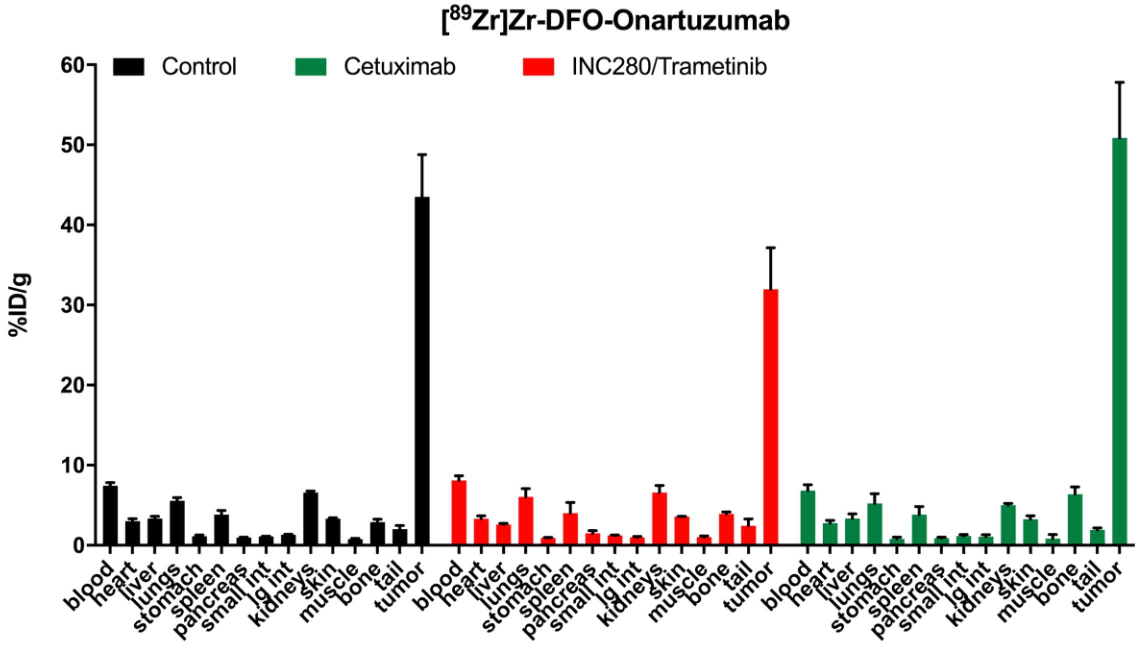

Supplemental Figure 6. Biodistribution at 120 h p.i. of [^{89}Zr]Zr-DFO-onartuzumab in NSG mice bearing subcutaneous PDX RCC and treated with saline (control), cetuximab or a combination of INC280 and trametinib (labeled as INC280/ trametinib). Cetuximab was intravenously administered (50 mg/kg of mice) twice a week for 10 days. INC280 (10 mg/kg of mice) and trametinib (1.5 mg/kg of mice) were orally administered daily for 10 days. [^{89}Zr]Zr-DFO-onartuzumab (2.7 MBq, 15μg) was administrated by tail vein injection on day 10 (*see Fig.* ***SI 4***). Biodistribution studies were performed at 120 h p.i. of [^{89}Zr]Zr-DFO-onartuzumab.

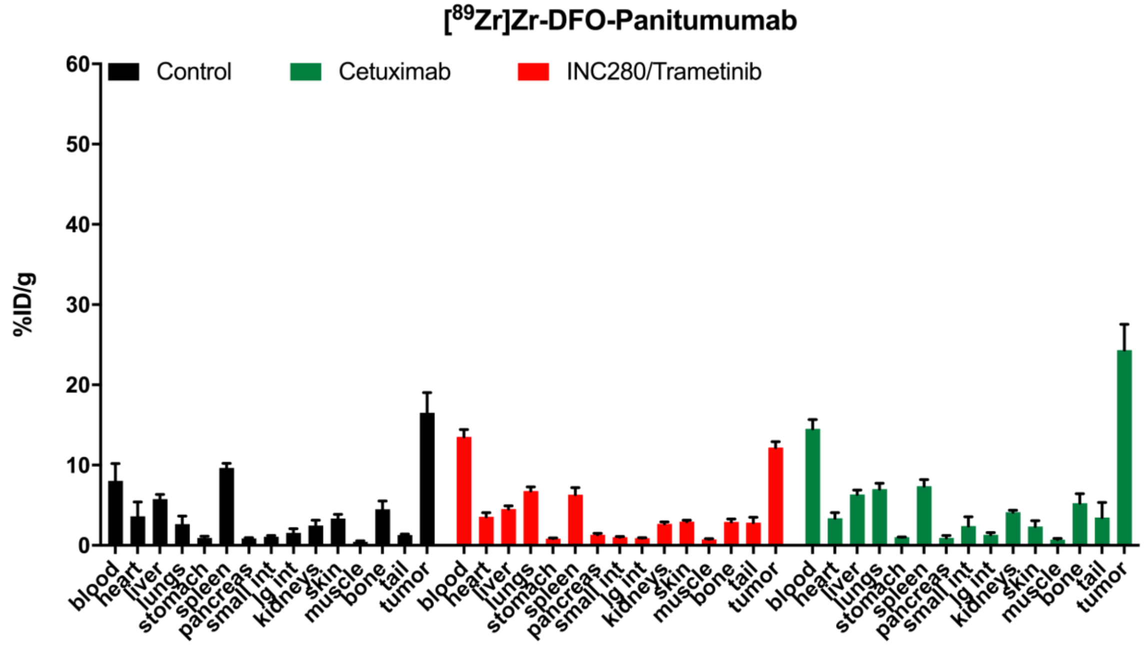

Supplemental Figure 7. Biodistribution at 120 h p.i. of [[^{89}Zr]Zr-DFO-panitumumab in NSG mice bearing subcutaneous PDX RCC and treated with saline (control), cetuximab or a combination of INC280 and trametinib (labeled as INC280/ trametinib). Cetuximab was intravenously administered (50 mg/kg of mice) twice a week for 10 days. INC280 (10 mg/kg of mice) and trametinib (1.5 mg/kg of mice) were orally administered daily for 10 days. [^{89}Zr]Zr-DFO-panitumumab (11.0 MBq, 50 µg protein) was administered by tail vein injection on day 10 (*see Fig. **SI 4***). Biodistribution studies were performed at 120 h p.i. of [^{89}Zr]Zr-DFO-panitumumab.

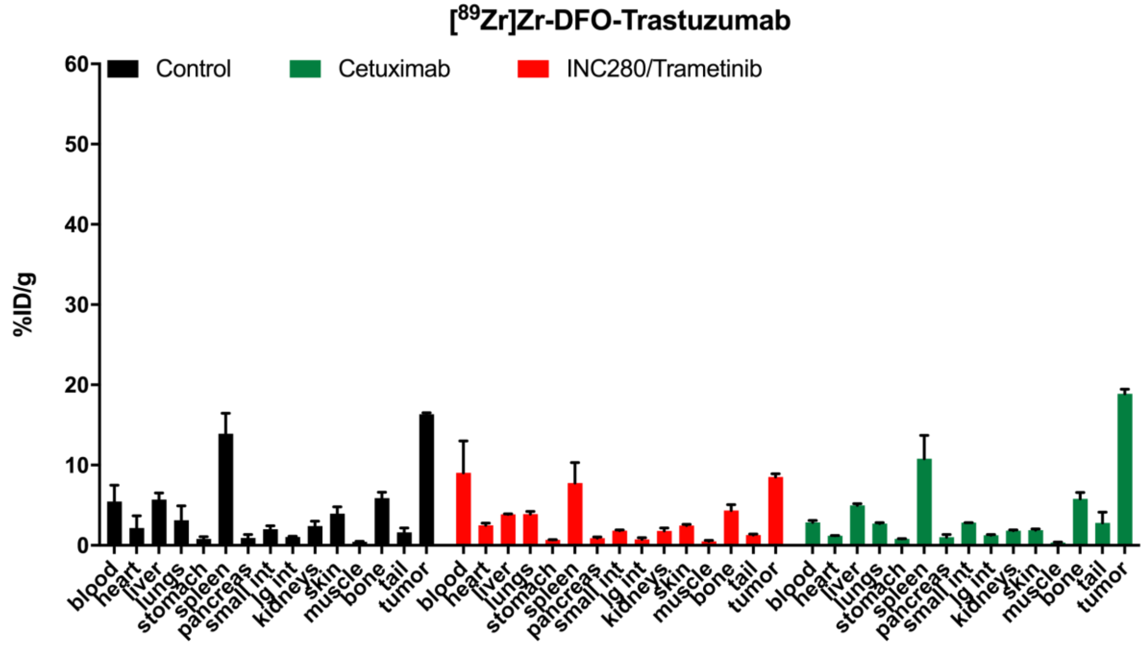

Supplemental Figure 8. Biodistribution at 120 h p.i. of [^{89}Zr]Zr-DFO-trastuzumab in NSG mice bearing subcutaneous PDX RCC and treated with saline (control), cetuximab or a combination of INC280 and trametinib (labeled as INC280/ trametinib). Cetuximab was intravenously administered (50 mg/kg of mice) twice a week for 10 days. INC280 (10 mg/kg of mice) and trametinib (1.5 mg/kg of mice) were orally administered daily for 10 days. [^{89}Zr]Zr-DFO-trastuzumab (8.14 MBq, 80 µg protein) was administered by tail vein injection on day 10 (*see Fig. **SI 4***). Biodistribution studies were performed at 120 h p.i. of [^{89}Zr]Zr-DFO-trastuzumab.
